# The Effect of Religion on Psychological Resilience in Healthcare Workers During the Coronavirus Disease 2019 Pandemic

**DOI:** 10.3389/fpsyg.2021.628894

**Published:** 2021-03-11

**Authors:** Mei-Chung Chang, Po-Fei Chen, Ting-Hsuan Lee, Chao-Chin Lin, Kwo-Tsao Chiang, Ming-Fen Tsai, Hui-Fang Kuo, For-Wey Lung

**Affiliations:** ^1^Department of Nursing, Calo Psychiatric Center, Pingtung, Taiwan; ^2^Department of Psychology, Calo Psychiatric Center, Pingtung, Taiwan; ^3^Department of Nursing, Jianren Hospital, Kaohsiung, Taiwan; ^4^Department of Medicine, Kaohsiung Armed Forces General Hospital Gangshan Branch, Kaohsiung, Taiwan; ^5^Department of Nursing, Kaohsiung Armed Forces General Hospital Gangshan Branch, Kaohsiung, Taiwan; ^6^Department of Medicine, Calo Psychiatric Center, Pingtung, Taiwan; ^7^Graduate Institute of Medical Science, National Defense Medical Center, Taipei, Taiwan; ^8^International Graduate Program of Education and Human Development, National Sun Yat-sen University, Kaohsiung, Taiwan; ^9^Institute of Education, National Sun Yat-sen University, Kaohsiung, Taiwan

**Keywords:** COVID-19, health care workers, mental distress, resilience, religion

## Abstract

**Background:** Healthcare workers in the front line of diagnosis, treatment, and care of patients with coronavirus disease 2019 (COVID-19) are at great risk of both infection and developing mental health symptoms. This study aimed to investigate the following: (1) whether healthcare workers in general hospitals experience higher mental distress than those in psychiatric hospitals; (2) the role played by religion and alexithymic trait in influencing the mental health condition and perceived level of happiness of healthcare workers amidst the stress of the COVID-19 pandemic; and (3) factors that influence the resilience of healthcare workers at 6 weeks' follow-up.

**Methods:** Four-hundred and fifty-eight healthcare workers were recruited from general and psychiatric hospitals, and 419 were followed-up after 6 weeks. All participants filled out the 20-item Toronto Alexithymia Scale, five-item Brief-Symptom Rating Scale, and the Chinese Oxford Happiness Questionnaire.

**Results:** Under the stress of the COVID-19 pandemic, 12.3% of frontline healthcare workers in general hospitals reported having mental distress and perceived lower social adaptation status compared with those working in psychiatric hospitals. Christians/Catholics perceived better psychological well-being, and Buddhists/Taoists were less likely to experience mental distress. The results at 6 weeks of follow-up showed that the perceived lower social adaptation status of general hospital healthcare workers was temporary and improved with time. Christian/Catholic religion and time had independent positive effects on psychological well-being; however, the interaction of Christian/Catholic religion and time had a negative effect.

**Conclusions:** Collectivism and individualism in the cultural context are discussed with regard to alexithymic trait and Buddhist/Taoist and Christian/Catholic religious faiths. Early identification of mental distress and interventions should be implemented to ensure a healthy and robust clinical workforce for the treatment and control of the COVID-19 pandemic.

## Introduction

Healthcare workers in the front line of diagnosis, treatment, and care of patients with confirmed and suspected coronavirus disease 2019 (COVID-19) are at great risk of both infection and developing mental health issues (Bfefferbaum and North, 2020; Chen et al., 2020; Neto et al., 2020). Furthermore, physicians and nurses who provided direct care for patients with COVID-19 experienced significantly more depression, anxiety, and insomnia and higher psychological distress than healthcare professionals who did not provide direct care (Lai et al., 2020). The 2003 severe acute respiratory syndrome (SARS) outbreak showed that healthcare workers were under tremendous pressure from the risk of being infected (Lu et al., 2006). Similar results have been found during the COVID-19 outbreak (Kang et al., 2020). One review showed that 71.5% of healthcare providers reporting mild to severe symptoms of post-traumatic stress disorder (Carmassi et al., 2020). Another systematic review and meta-analysis showed that the prevalence of stress was 45%, depression 24.3%, and anxiety 25.8% in frontline healthcare workers caring for patients with COVID-19 (Salari et al., 2020). Resilience is the ability of an individual to withstand setbacks, adapt positively, and bounce back from adversity (Luthar and Cicchetti, 2001). Enhancing factors that may contribute to psychological resilience during the pandemic period, thus ensuring a physically and mentally healthy clinical workforce, are important for the treatment and control of the pandemic.

Emotion and stress coping strategies are vital for healthcare professionals during the pandemic period and may subsequently influence their mental condition and well-being. Alexithymia is a trait of deficiency in the ability to identify, differentiate, verbalize, and communicate feelings (Taylor, 1994). Given that emotions serve communication and social functions in informing others about our thoughts and intentions, they coordinate our social interaction and self-regulation. Alexithymia has been found to be highly associated with mental health condition and psychiatric disorders (Chen et al., 2011). Therefore, we are interested in the role played by alexithymic trait when healthcare workers are under the stress of the COVID-19 pandemic.

Religion has an impact on well-being when people encounter stressful situations and adversities in life (Park, 2005). It acts as an important philosophical orientation that influences how people understand the world and comprehend reality and suffering (Pargament, 1997). Park (2005) found a pathway relationship for religion as a meaning-making coping (positive reappraisal coping), which leads to stress-related growth. Furthermore, religion is associated with active coping and positive reframing coping strategies, and electroencephalography activity was observed in the theta frequency band in the right hemisphere in association with religious coping (Imperatori et al., 2020). A study in Italy found that people personally close to the effects of COVID-19 turned toward religion as a coping strategy (Molteni et al., 2020). However, a study in China did not find an association between religiousness and perceived stress in healthcare professionals during the COVID-19 pandemic (Babore et al., 2020). An empirical study also found that only one out of 11 studies reviewed displayed a significant association between religion and traumatic stress (Chen and Koenig, 2006). Therefore, the factor of religion, and how different religions may influence the appraisal and psychological resilience of these healthcare workers, is also investigated.

Therefore, this study aimed to investigate the following: (1) whether healthcare workers working in general hospitals experience higher mental distress, which may influence their psychological well-being and social adaptation status, compared with workers in psychiatric hospitals; (2) the role played by religion and alexithymic trait in influencing the mental health condition and perceived level of happiness of healthcare workers amidst the stress of the COVID-19 pandemic; and (3) factors that influence the resilience of healthcare workers at 6 weeks of follow-up.

## Materials and Methods

### Participants

Healthcare workers from three hospitals in Southern Taiwan were convenience sampled from February 16 to 20, 2020, and followed-up 6 weeks later (April 6 to 13). Two of the hospitals are general hospitals and one is a psychiatric hospital. All three hospitals were in Southern Taiwan, so that culture and beliefs were similar, for the purpose of neighborhood control. The superintendent and head of department of the two general hospitals were invited to cooperate in this study. After they agreed to cooperate, a research assistant from the psychiatric hospital contacted each participant and explained the purpose of this study, and if they agreed to enroll in the study, the survey was distributed for them to fill out. A total of 458 healthcare workers were recruited at the first stage, 274 from the first general hospital, 98 from the second general hospital, and 84 from the psychiatric hospital. Four hundred and nineteen (91.5%) were recruited at follow-up, with 257 from the first general hospital, 88 from the second general hospital, and 74 from the psychiatric hospital. Owing to the shifts of healthcare workers, not all participants were able to participate in the 6-week follow-up. Chi-square analysis of general and psychiatric hospital healthcare workers completing the first and second surveys resulted in a value of 1.517 (*p* = 0.218), showing that differences in intention to participate in the research were not statistically significant. The three hospitals have a total of 583 employees; thus, our study had a response rate of 78.6%. The procedures performed in this study were approved by the Institutional Review Board of Kaohsiung Armed Forces General Hospital (approval number: KAFGH 109-008), in Kaohsiung, Taiwan, and are in accordance with the 1964 Helsinki Declaration and its later amendments or comparable ethical standards.

### Measurement

All information collected was from participants' self-report. The participants completed a demographic information sheet (which included a question about their religion), the 20-item Toronto Alexithymia Scale (TAS-20), five-item Brief-Symptom Rating Scale (BSRS-5), and the Chinese Oxford Happiness Questionnaire; all the above surveys were in Chinese, and is also included in the Supplementary material. The BSRS, the happiness scale, and TAS-20 were also completed at 6 weeks of follow-up.

#### Religion

The Executive Yuan, Taiwan reported 33 main religions in Taiwan, with 35% of the population reported to be Buddhist and 33% Taoist, followed by 2.6% Protestant Christian and 1.3% Catholic Christian (Office of International Religious Freedom, 2019). On the other hand, 20% of the population was reported to have no religious faith (Office of International Religious Freedom, 2019). Therefore, the demographic information sheet included the religious faith choices of “Buddhism,” “Taoism,” “Christian (Protestant),” “Catholic,” and “others.”

#### Alexithymia

The TAS-20 self-report inventory was used to measure the level of alexithymia in these healthcare workers. The TAS-20 measures alexithymia in three dimensions: difficulty identifying feelings, difficulty describing feelings, and externally oriented thinking (Taylor, 1994). The Chinese version of the TAS-20 has shown good internal consistency (Lin and Chan, 2003). Furthermore, an empirically derived cut off score of 61 is used to identify individuals who are considered to have alexithymia (Taylor et al., 1997). Therefore, the cutoff of 61 was used to categorize the healthcare workers into two groups: those with alexithymic trait (TAS-20 scores ≥61) and those without (TAS-20 scores <60).

#### Mental Health Condition

The BSRS-5 is a short and efficient screening instrument aimed to screen for psychiatric illnesses in the community, as well as in general medical and psychiatric settings. It measures the five symptom domains of anxiety, depression, hostility, interpersonal sensitivity/inferiority, and insomnia. The Chinese version of the BSRS-5 has been shown to be valid to screen for mental health conditions of psychiatric inpatients, general medical patients, and community residents in Taiwan (Lung and Lee, 2008). Using the histogram graphical method, the cutoff of 10/11 was found most appropriate for this group of healthcare workers. Therefore, the participants were separated into two groups, those scoring ≥10 being considered as those with greater mental health distress.

#### Happiness

The translated and culturally modified seven-item Chinese Oxford Happiness Questionnaire was used to measure the self-perceived level of happiness of the healthcare workers. The Chinese Oxford Happiness Questionnaire measures happiness in the two dimensions of social adaptation status (SAS) and psychological well-being (PWB) and has shown good psychometric properties in measuring the level of happiness of community participants in Taiwan (Lung and Shu, 2020).

### Statistical Analysis

Descriptive analysis was used to analyze the demographic information and TAS-20, BSRS-5, and Chinese Oxford Happiness Questionnaire scores of the healthcare workers at the beginning of the pandemic and at 6 weeks of follow-up.

Structural equation modeling (SEM) was used to analyze the baseline dataset, collected at the beginning of the pandemic. Using SEM, graphical pathway relationships among religion, alexithymic trait, mental distress, psychological well-being, and social adaptation status of healthcare workers in general and psychiatric hospitals were investigated. The SEM uses the χ^2^ fit test to investigate the overall fit of the model; non-significant χ^2^ values (*p* > 0.05), adjusted goodness-of-fit indices (AGFI) >0.9, and root mean square error of approximation (RMSEA) <0.05 indicated that the model adequately described the observed data.

Generalized equation estimation (GEE) analysis, most suitable for measurement of repeated data, was used to investigate which factors affected the psychological resilience of the healthcare workers at 6 weeks of follow-up. Parsimonious results were presented in the SEM and GEE analysis, which means that only statistically significant pathways/variables were presented, and all paths with *p* > 0.05 were deleted.

Descriptive analysis and GEE analysis were carried out using Statistical Package for the Social Sciences (SPSS) 26.0 for Windows software (SPSS Inc., Chicago, USA). SEM was analyzed using the Analysis of a Moment Structures 26.0 statistical software package (SPSS Inc., Chicago, USA).

## Results

The baseline and follow-up distributions of the sociodemographic data and TAS-20, BSRS-5, and Chinese Oxford Happiness Questionnaire scores of those who work in general and psychiatric hospitals are shown in Table 1.

**Table 1:** Baseline socio-demographic and clinical characteristics of healthcare workers (*N* = 458).

	**Beginning of pandemic**		**6 weeks' follow-up**	
**Variable**	**General hospital** **(*n* = 374)**	**Psychiatric hospital** **(*n* = 84)**	**General hospital** **(*n* = 345)**	**Psychiatric hospital** **(*n* = 74)**
	***n* (%)**	***n* (%)**	***n* (%)**	***n* (%)**
Sex				
Male	31 (8.3)	18 (21.4)	24 (7.0)	15 (20.3)
Female	343 (91.7)	66 (78.6)	321 (93.0)	59 (79.7)
Religion				
Buddhist and Taoist	238 (63.6)	52 (61.9)	230 (66.7)	49 (66.2)
Christian and Catholic	33 (8.8)	17 (20.7)	32 (9.3)	11 (14.9)
Others	103 (27.5)	15 (17.9)	83 (24.1)	14 (18.9)
TAS-20 ≥ 61	34 (9.1)	5 (6.0)	39 (11.3)	5 (6.8)
BSRS-5 ≥ 10	46 (12.3)	3 (3.6)	45 (13.0)	4 (5.4)
**Variable (range)**	**mean (SD)**	**mean (SD)**	**mean (SD)**	**mean (SD)**
Age (20–68)	39.18 (9.74)	40.59 (11.12)	39.22 (9.62)	40.29 (11.16)
TAS-20 total score (20–80)	48.71 (9.60)	44.70 (11.47)	48.75 (9.56)	45.03 (12.03)
BSRS-5 total score (0–18)	4.58 (3.82)	2.11 (2.96)	4.77 (3.79)	2.35 (3.02)
Happiness scale				
Social adaptation status (6–16)	11.99 (1.98)	13.14 (1.96)	12.14 (1.94)	12.92 (2.01)
Psychological well-being (3–12)	8.16 (1.87)	8.62 (2.06)	8.13 (1.85)	8.70 (1.96)

*TAS-20, Toronto Alexithymia Scale; BSRS-5, five-item Brief-Symptom Rating Scale; SD, standard deviation*.

### SEM: First Stage Dataset

**Figure 1:**
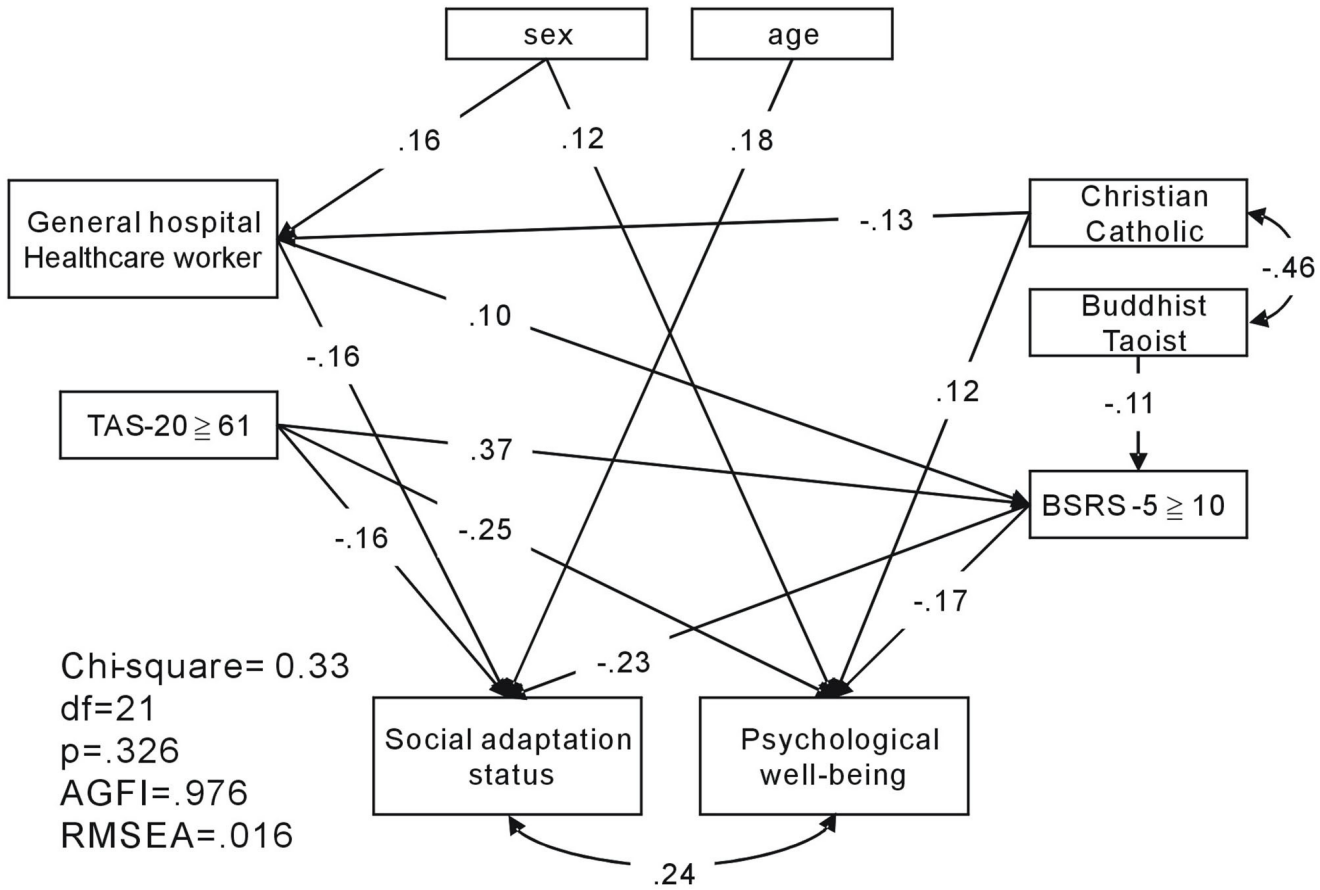
Structural equation model of religion, alexithymic trait, mental distress, and level of happiness of healthcare workers in general and psychiatric hospitals. AGFI, adjusted goodness-of-fit; RMSEA, root mean square error of approximation.

SEM was used to investigate the graphical pathway relationships of religious faith (Buddhism/Taoism and Christianity/Catholicism), alexithymic trait, mental distress, psychological well-being, and social adaptation status of healthcare workers in general and psychiatric hospitals. The model resulted in *p* = 0.326 (>0.05), AGFI = 0.976 (>0.9), and RMSEA = 0.016 (<0.08), implying that the null model approximates the real structure, as shown in Figure 1. Overall, females perceived better psychological well-being than males (β = 0.12, *p* = 0.004), and those who were older reported better social adaption status than those who were younger (β = 0.18, *p* < 0.001). Those who worked in general hospitals, compared with those working at the psychiatric hospital, were more likely to experience mental distress (β = 0.10, *p* = 0.027) and lower social adaptation (β = −0.16, *p* < 0.001). Healthcare workers who were Christian/Catholic perceived better psychological well-being (β = 0.12, *p* = 0.003), and Buddhists/Taoists were less likely to experience mental distress (β = −0.11, *p* = 0.009), which would indirectly increase their perceived level of happiness (social adaptation status: β = −0.23, *p* < 0.001; psychological well-being: β = −0.17, *p* < 0.001). Healthcare workers that had alexithymic trait experienced greater mental distress (β = 0.37, *p* < 0.001) and perceived a lower level of happiness (social adaptation status: β = −0.16, *p* < 0.001; psychological well-being: β = −0.25, *p* < 0.001).

### GEE: Follow-Up Analysis

GEE was used to investigate which factors were associated with psychological well-being and social adaptation status of these healthcare workers during the pandemic at 6 weeks of follow-up. As Table 2 shows, those who had lower alexithymic level (TAS-20), lower mental distress level (BSRS-5), or were Christian/Catholic (β = −0.09, *p* < 0.001; β = −0.08, *p* < 0.001; β = 0.69, *p* = 0.006) showed a higher level of psychological well-being. However, the interaction of time and Christian/Catholic religion showed that those who were Christian/Catholic had lower psychological well-being over time (β = −0.92, *p* < 0.001).

**Table 2:** Generalized equation estimation results of the factors associated with the level of happiness of healthcare workers at follow-up.

**Dependent variable**	**Independent variable**	**ß**	**S.E**.	**95% C.I**.	***p***
Psychological well-being	Time	0.14	0.081	−0.02 to 0.30	0.078
	Alexithymic level (TAS-20)	−0.09	0.007	−0.10 to −0.07	<0.001
	Mental distress level (BSRS-5)	−0.08	0.020	−0.12 to −0.04	<0.001
	Christian and Catholic	0.69	0.249	0.20 to 1.18	0.006
	Christian/Catholic × time	−0.92	0.261	−1.43 to −0.41	<0.001
Social adaptation status	Time	−0.36	0.182	−0.71 to 0.00	0.050
	Works at general hospital	−0.50	0.201	−0.89 to −0.10	0.013
	General hospital × time	0.74	0.208	0.33 to 1.15	<0.001
	Age	0.03	0.006	0.01 to 0.04	<0.001
	Alexithymic level (TAS-20)	−0.06	0.007	−0.07 to −0.04	<0.001
	Mental distress level (BSRS-5)	−0.16	0.018	−0.20 to −0.12	<0.001

*Multiplication sign (×) indicates interaction. TAS-20, Toronto Alexithymia Scale; BSRS-5, five-item Brief-Symptom Rating Scale; SE, standard error; CI confidence interval*.

Regarding social adaptation status, after 6 weeks, those who worked at general hospitals showed worse social adaptation status (β = −0.36, *p* = 0.050; β = −0.50, *p* = 0.013). However, the interaction of time and general hospital working showed, over time, that those who worked in general hospitals had better social adaptation status (β = 0.74, *p* < 0.001). Furthermore, those who were older, had lower alexithymic trait, and/or lower mental distress level had better social adaptation status (β = 0.03, *p* < 0.001; β = −0.06, *p* < 0.001; β = −0.16, *p* < 0.001).

## Discussion

Our study found that, under the stress of the COVID-19 pandemic, 12.3% of frontline healthcare workers in general hospitals reported having mental distress, which is significantly higher than the 3.6% reported by healthcare workers in the psychiatric hospital (*p* = 0.018). The SEM showed that medical staff at general hospitals not only experienced greater mental distress but also perceived lower social adaptation status when compared with those working in psychiatric hospital. We also studied the roles of emotion and religious faith in coping with the stress of the pandemic. Although there were no differences in alexithymia (TAS-20 ≥ 61) between those working in general and psychiatric hospitals, healthcare workers that had alexithymic trait were more likely to experience mental distress and reported a lower level of happiness (social adaptation status and psychological well-being). Furthermore, Christians or Catholics perceived better psychological well-being, and Buddhists or Taoists were less likely to experience mental distress, which would indirectly increase their level of happiness (psychological well-being and social adaptation status). At follow-up 6 weeks later, those of Christian/Catholic faith had developed lower psychological well-being over time, but healthcare workers who worked at general hospitals perceived better social adaptation status over time.

Healthcare workers in general hospitals reported having higher levels of mental distress and felt less socially adapted than those working in psychiatric hospital. This is similar to a research in China showing that frontline healthcare workers, who have to directly diagnose, treat, and take care of patients with COVID-19, were at higher risk of psychological symptoms of depression, anxiety, insomnia, and distress (Lai et al., 2020). In a broader context, a study in Peru also found a “ripple effect,” in that healthcare workers who were geographically further from the epicenter of the COVID-19 outbreak experienced less anxiety and mental distress (Yáñez et al., 2020). Furthermore, healthcare workers may experience isolation, distress caused by concerns about their own health, and the possibility of putting the health of their family and friends at risk (Stojanov et al., 2020). Consequently, frontline medical personnel will feel less socially adapted compared with second-line medical personnel (Master et al., 2020). Fortunately, our follow-up results showed that the social adaptation status of general hospital healthcare workers increased at 6 weeks' follow-up.

With regard to the ability to identify, express, and distinguish emotion from physical sensations of emotional arousal, no differences in alexithymia (TAS-20 ≥ 61) were found between those working in general and psychiatric hospitals. Nevertheless, healthcare workers who had alexithymic trait were more likely to experience mental health distress and a lower level of happiness (social adaptation status and psychological well-being). After 6 weeks, healthcare workers who had higher alexithymic trait still showed a lower level of happiness. Given that emotions serve as a communication and social function to inform others about our thoughts and intentions, they coordinate our social interaction and self-regulation. Therefore, alexithymia is associated with problems with emotional regulation and adaptive coping (Parker et al., 2001) and, subsequently, with happiness and psychological well-being (Páez et al., 2013). Consequently, at 6 weeks' follow-up, we found that those with lower alexithymic trait, meaning healthcare workers who were more aware of their own emotions, being able to distinguish and express their emotions, showed better psychological resilience under the stress of the pandemic. The East Asian culture of collectivism places more emphasis on emotional restraint, the importance of attending to social cues, and the maintenance of social harmony and less emphasis on individual emotional experience and expression (as characterized by the individualist western cultural context) (Ryder et al., 2008). Under such a cultural context, Asian parents are more controlling, restrictive, and authoritarian and show less positive emotion toward their children (Morelen and Thomassin, 2013). The children grow up in a family environment in which they suppress expression of their feelings and have higher levels of alexithymia (Berenbaum and James, 1994). Therefore, studies have found that children in Taiwan show slower emotional development (Lung et al., 2020), and Asian Americans also have higher levels of alexithymia when compared with European American college students (Le et al., 2002). Similarly, a study in China also found alexithymia to be a mediator between COVID-19 exposure and mental health problems in university students (Tang et al., 2020). This is important, because alexithymic trait can increase the risk of mental health conditions and psychiatric disorders (Chen et al., 2011). The results of these studies and our results all show the importance of including the cultural context in the investigation of emotional development and expression.

In addition to emotional coping strategies, our study also showed that religious faith has an impact on the mental health and level of happiness of healthcare workers. Buddhists or Taoists were less likely to experience mental distress, and this would indirectly increase their level of happiness (psychological well-being and social adaptation status). Interestingly, our follow-up data found that, while Christian/Catholic religion and time had independent positive effects on psychological well-being, the interaction of Christian/Catholic faith and time had a negative effect on psychological well-being. This means that, although those with Christian/Catholic religious faith showed better psychological well-being, over time, their recovery rate was slower compared with those of other religious faiths. Religion can be seen as a meaning-making coping system, which influences subjective well-being and the way in which we cope with traumatic stressful events in life (Park, 2005). Coulombe et al. (2020) found that the process of integrating psychological, social, and systemic factors increases the resilience of mental health and well-being during the pandemic. Furthermore, the practice of religion, expressions of faith, and cultivation of spirituality can augment our physical and psychological resilience (Levin, 2020). The relationship of religion and culture is also intertwined (Cohen et al., 2016). Eastern collectivism culture shapes the development of Eastern religions (including Buddhism and Taoism), and religion in turn can influence the cultural development of nations (such as Christianity and the culture of the USA) (Cohen and Hill, 2007). Similar to the discussion about cultural context and emotion, religious cultures differ in the emotional state they value and encourage their practitioners to feel (Tsai et al., 2007). Christianity values high-arousal positive states (such as excitement), whereas Buddhism values low-arousal positive states (such as calm) (Tsai et al., 2007). Our dataset also showed Buddhism to be positively associated with alexithymia and Christianity to be negatively associated with alexithymia; however, the association was not statistically significant (data not shown). Buddhism advocates a practice that emphasizes a balance between asceticism and self-indulgence and avoids extremes in life in order to reach calm detachment. Buddhist values of tolerance, non-violence, respect for the individual, and a belief in the fundamental spiritual equality of all humans are continued in interpersonal relationships under collectivism cultural values (Konsky et al., 2000). In contrast, Christians believe in a personal soul and internal attributes of a person who behaves in the way they do because of their traits or disposition and their personal faith in God (Cohen et al., 2016). Therefore, it is understandable that when looking at the association of Buddhism/Taoism and Christianity/Catholicism with happiness dimensions of psychological well-being and social adaptation status, Christianity/Catholicism impacts the dimension of psychological well-being (which is related to the individual-oriented aspect of happiness) and Buddhism/Taoism is indirectly associated with both psychological well-being and social adaptation status (social-oriented aspects of happiness) (Lung and Shu, 2020).

A limitation of this study was that the healthcare workers recruited in this study were all from Southern Taiwan. A study in Peru showed that healthcare workers in the epicenter of the pandemic showed higher psychological distress than those further from the epicenter (Yáñez et al., 2020). Given that the epicenter of the pandemic was located in the northern part of Taiwan, the psychological distress and well-being of healthcare workers in different geological regions and the generalizability of our results need to be further investigated.

Our study shows that frontline healthcare workers in general hospitals reported a higher rate of mental distress and a lower level of social adaptation than second-line healthcare workers. Fortunately, the perceived lower social adaptation status of general hospital healthcare workers was temporary and improved with time. Religious faith can help individuals to cope with the stress of the pandemic, with Christianity/Catholicism elevating psychological well-being and Buddhism/Taoism decreasing mental distress and indirectly elevating the level of happiness of these healthcare workers. However, at 6 weeks' follow-up, the psychological well-being recovery rate of Christians/Catholics was slower than that of other religious faiths. Further follow-up will help us to clarify whether it is daily life stress or threat from the pandemic that impacts the mental health state of these participants (Lung et al., 2009). Peer support, supportive therapy, and early identification of mental distress and interventions should be implemented (Banerjee, 2020) to alleviate mental health conditions and decrease the feeling of isolation and social maladaptation of these medical personnel. Addressing the mental health conditions of medical workers is important to ensure a healthy and robust clinical workforce and for the treatment and control of the COVID-19 pandemic (Banerjee, 2020).

## Data Availability Statement

The raw data supporting the conclusions of this article will be made available by the authors, without undue reservation.

## Ethics Statement

The studies involving human participants were reviewed and approved by Kaohsiung Armed Forces General Hospital (approval number: KAFGH 109-008). The patients/participants provided their written informed consent to participate in this study.

## Author Contributions

M-CC and F-WL designed the study. M-CC, C-CL, K-TC, M-FT, and H-FK overlooked the sampling and experimental procedures. P-FC, T-HL, and F-WL undertook the statistical analysis and interpreted the analysis. P-FC wrote the first draft of the manuscript. All authors contributed to and have approved the final manuscript.

## Conflict of Interest

The authors declare that the research was conducted in the absence of any commercial or financial relationships that could be construed as a potential conflict of interest.
