# Supplementary material for: The Effect of Religion on Psychological Resilience in Healthcare Workers During the Coronavirus Disease 2019 Pandemic
Source: Front Psychol. 2021 Mar 11;12:628894. doi: 10.3389/fpsyg.2021.628894 (PMC7991302; doi:10.3389/fpsyg.2021.628894)
Supplement: Supplementary file 1 [file Table_1.DOCX]

**Supplementary material**

20-item Toronto Alexithymia Scale (TAS-20)

1. I am often confused about what emotion I am feeling.
2. It is difficult for me to find the right words for my feelings.
3. I have physical sensations that even doctors don’t understand.
4. I am able to describe my feelings easily.
5. I prefer to analyze problems rather than just describe them.
6. When I am upset, I don’t know if I am sad, frightened, or angry.
7. I am often puzzled by sensations in my body.
8. I prefer to analyze problems rather than just describe them.
9. I have feelings that I can’t quite identify.
10. Being in touch with emotions is essential.
11. I find it hard to describe how I feel about people.
12. People tell me to describe my feelings more.
13. I don’t know what’s going on inside me.
14. I often don’t know why I am angry.
15. I prefer talking to people about their daily activities rather than their feelings.
16. I prefer to watch “light” entertainment shows rather than psychological dramas.
17. It is difficult for me to reveal my innermost feelings, even to close friends.
18. I can feel close to someone, even in moments of silence.
19. I find examination of my feelings useful in solving personal problems.
20. Looking for hidden meanings in movies or plays distracts from their enjoyment.

Five-item Brief-Symptom Rating Scale (BSRS-5)

1. Trouble falling asleep.
2. Feeling tense or high-strung.
3. Feeling irritable or angry.
4. Feeling down, depressed.
5. Feeling inferior to others.

Chinese Oxford Happiness Questionnaire

1. I find beauty in some things.
2. I don’t feel particularly pleased with the way I am.
3. I feel that life is very rewarding.
4. I am well satisfied about everything in my life.
5. I don’t think I look attractive.
6. I can fit in everything I want to.
7. I do not have particularly happy memories of the past.
